# Microbiological Spectrum of Neutropenic Sepsis in Cancer Patients Admitted to a Tertiary Health Care Centre

**DOI:** 10.7759/cureus.43898

**Published:** 2023-08-22

**Authors:** Kevin R John, Arun Warrier, Anup Warrier

**Affiliations:** 1 Medical Oncology, Aster Medcity, Cochin, IND; 2 Infectious Diseases, Aster Medcity, Cochin, IND

**Keywords:** hematological malignancies, antibiotic susceptibility, microbiological spectrum, febrile neutropenia, cancer, anti-microbial resistance, neutropenic sepsis

## Abstract

Objective: To examine the microbiological profile, sensitivity of organisms, treatment and outcomes of in-patients suffering from febrile neutropenia in a tertiary healthcare centre.

Methods: Data was obtained from the Electronic Medical Health records in Aster Medcity, Cochin, IND. The study population included adult patients undergoing treatment for hematologic malignancies or solid tumors in the hospital between January 2021 and March 2023. Febrile neutropenia episodes were identified based on (1) absolute neutrophil count ≤1500 mm^3^, (2) at least a single recorded oral temperature of >38.0^∘^C (100.4^∘^F) sustained over a one-hour period. Febrile neutropenia consequences included ICU admission, length of ICU admission, and mortality.

Results: Total 115 cases of febrile neutropenia were identified in the time period from January 2021 to March 2023. Organisms were isolated from 43% of all the cultures taken. The most common organism isolated was *Klebsiella pneumoniae* (32.81%), followed by *Escherichia coli* (29.69%) and *Pseudomonas aeruginosa* (10.94%). Other organisms that were also isolated were *Candida albicans* (3.13%), *Aeromonas hydrophilia*, *Acinetobacter baumannii*, *Burkholderia cepacia*, *Enterobacter cloacae*, *Enterococcus faecium*, *Staphylococcus epidermidis*, *Staphylococcus hemolyticus*, *Streptococcus* spp, and one case of *Ralstonia mannitolytica*. Multi-drug resistance (MDR) was seen in 33% of isolates and extensive-drug resistance was seen in 19% of isolates. *E. coli* showed the highest prevalence of antibiotic resistance with 68% growing MDR isolates and 16% growing XDR isolates. ICU stay was required in 34% of patients with a median duration of stay of three days. A mortality rate of 16.52% was seen, with 17.11% in hematological malignancies and 15.38% in solid tumors.

Conclusions: This study showed an increasing prevalence of Gram-negative bacterial infection in patients with febrile neutropenia. It also shows a high prevalence of antibiotic resistance in microbes in febrile neutropenia. Larger multi-hospital studies are required to better understand the microbiological profile of febrile neutropenia and identify the developing antimicrobial resistance trends.

## Introduction

Cancer is currently one of the biggest challenges faced by modern medicine. While modern medicine has come a long way in treatment options for cancer, sometimes the cure may hit as hard as the disease. One of the most common side effects is a decrease in the count of cells involved in the body’s immune system. Sometimes the decrease in cell count becomes so significant, that the body becomes unable to mount a defense against external microorganisms, which may lead to an increased risk of infections.

The most commonly affected cells in this situation are the neutrophils. Neutrophils are the primary mediators of the rapid innate host defense against most bacterial and fungal pathogens that occurs before the complex humoral and lymphocyte cellular processes of acquired immunity can be brought to bear on an infection. Neutrophils help control bacterial infections with their rapid response before they can multiply in number [1].

Febrile neutropenia is defined as a decrease in neutrophil counts associated with a fever. Febrile neutropenia is considered an oncological emergency requiring admission and IV antibiotics, and if left untreated, can lead to severe infection complications and possible death [2]. Despite major advances in treatment and prevention, febrile neutropenia continues to be one of the most dangerous and frequent complications of cancer chemotherapy. It is also a major cause of mortality and morbidity among patients [3-10]. It also results in decreased efficacy resulting from delays and decreased doses of treatment [10,11].

Mortality from febrile neutropenia has decreased over the years but still remains significant. Mortality rates are around 5% in patients with solid tumors and as high as 11% in some hematological malignancies [3-6,12]. The prognosis is worst in patients with proven bacteremia [7-12]. Moreover, febrile neutropenia compromises the chemotherapy and treatment by resulting in dose reduction and/or delays in the administration of chemotherapy, thereby reducing the efficacy of treatment, patient survival, and quality of life [10].

Microbial etiology is often unknown at the initiation of treatment. In such scenarios, the choice of empiric antibiotic therapy should depend on locally prevalent pathogens and their sensitivities, potential sites of infection, and the cost of various regimens. At present, the Infectious Diseases Society of America (IDSA) and European Society for Medical Oncology (ESMO) guidelines are being followed by the most well-recognized treatment centers in India [12,13].

This study was designed to review microbial flora patterns, susceptibility, and important clinical variables among febrile neutropenic patients with solid tumors and hematological malignancies at Aster Medcity, Cochin, a tertiary health care center, to help decide future treatment for similar episodes.

Definitions

Fever in neutropenic patients is defined as a single oral temperature of >38.0∘C (100.4 °F) sustained over a one-hour period [13]. Neutropenia is usually diagnosed using a routine complete blood count (CBC), with the accompanying differential count being used to calculate the absolute number of neutrophils. Mild neutropenia is defined as an absolute neutrophil count (ANC) of less than 1,500 cells/mm^3^. Moderate neutropenia is defined as a count less than 1,000 cells/mm^3^ or that is expected to decrease below 500 cells/mm^3^ during the next 48 hours. Less than 500 cells/mm^3^ is considered to be severe neutropenia. An absolute neutrophil count of less than 100 cells/mm^3^ was considered profound neutropenia [13,14]. Pancytopenia was defined as a hemoglobin count of <9 g/dL, platelet counts <100,000/µL, and total leukocyte count (TLC) <4,000/µL [15].

Sensitive (S) implies that isolates are inhibited by the usually achievable concentrations of antimicrobial agents and minimum inhibitory concentrations (MICs) are below the usual breakpoints, Resistant (R) implies that isolates are not inhibited by the usually achievable concentrations of antimicrobial agents, and MICs are above the usual breakpoints. Multi-drug resistant (MDR) is defined as non-susceptibility to at least one agent in three or more antimicrobial categories. Extensively drug-resistant (XDR) is defined as non-susceptibility to at least one agent in all but two or fewer antimicrobial categories [16].

## Materials and methods

Materials and methods

This retrospective observational study was done at Aster MedCity, Cochin, India, during the period of January 2021 to March 2023. All patients in the Medical Oncology Department suffering from hematological malignancies and solid tumors were analyzed for the diagnosis of febrile neutropenia. The study contained all instances of admission under Medical Oncology with a diagnosis of febrile neutropenia. These instances were identified using the International Classification of Disease, 9th edition, Clinical Modification (ICD-9-CM) code system [17]. A total of 283 cases of febrile neutropenia admissions were identified. The study included only patients ≥18 years, and 67 pediatric cases were identified and excluded.

Febrile neutropenia episodes were identified based on ANC <1500 cell/mm^3^ and evidence of fever by a recorded temperature of >100.4°F [13]. The usage of antibiotics was based on standard hospital protocols. The study's inclusion criteria were patients ≥18 years, satisfying the criteria for febrile neutropenia as defined above. Exclusion criteria were patients with diagnoses of non-malignant conditions, patients with co-morbid HIV or primary immunodeficiencies, and pregnant patients. Using the inclusion and exclusion criteria, 115 cases of febrile neutropenia involving 89 patients were identified. Laboratory confirmation of neutropenia was done by using the hospital lab reports. Microbial identification and antimicrobial susceptibility testing by MIC determination was performed by Vitek 2 compact (bioMérieux SA, France) as per Clinical and Laboratory Standards Institute (CLSI) guidelines [18]. Sensitive (S) implies that isolates are inhibited by the usually achievable concentrations of antimicrobial agents and MICs are below the usual breakpoints, Resistant (R) implies that isolates are not inhibited by the usually achievable concentrations of antimicrobial agents, and MICs are above the usual breakpoints.

We reviewed the electronic medical records, nursing records, and laboratory findings for all patients with confirmed febrile neutropenia. Data was collected on age, sex, co-morbidities (diabetes mellitus, hypertension, cerebrovascular accidents, myocardial infarction, hypothyroidism, dyslipidemia, chronic kidney disease, liver disease), type of cancer, and chemotherapy regimen. Microbiological profile was collected using blood cultures and other. Treatment given, antibiotics used, and course in the hospital were obtained by going through the medical notes and nursing records. For recording the microbial infection in patients with multiple cultures taken, only the first culture that showed growth was considered. Subsequent cultures were included only if they showed the growth of a new organism.

Analyses were purely descriptive. Incidence, microbiological flora, treatment, and consequences of febrile neutropenia during the episode were reported using means, and frequencies. This study was presented to the Institutional Ethics Committee, Aster Medcity, Cochin, India, on April 12, 2023, and was approved with IEC number AM/EC/30S-2023.

## Results

Baseline characteristics

We identified 115 cases of febrile neutropenia in a total of 89 patients that met our inclusion criteria. The discrepancy was observed between the number of febrile neutropenia cases and the number of patients this can be explained, as some patients would have multiple instances of febrile neutropenia during the course of their treatment. Their baseline characteristics have been presented in Table 1. The age range was from 20 to 83 years, with a median age of 51.5 years. Of the total cases, 83 (72.17%) were males whereas 32 (27.83%) were females. The most common comorbidity was diabetes mellitus which was seen in 30 cases (26.09%), followed by hypertension, which was seen in 25 cases (21.74%). History of myocardial infarction was present in 13 cases (11.30%), chronic obstructive pulmonary disease was present in eight cases (6.96%), dyslipidemia was seen in seven cases (6.09%), hypothyroidism was associated with five cases (4.35%), atherosclerosis was present in four cases (3.48%), chronic kidney disease in three patients (2.61%), and atrial fibrillation was present in one case (0.87%).

**Table 1:** Demographic profile of the study population sample size (n)= 115

Variables		Counts	Percentage %
Age in years	Range	20-83	
	Mean±SD	51.5±18.16	
Gender	Male	83	72.17%
	Female	32	27.83%
Comorbidities	Diabetes	30	26.09%
	Hypertension	25	21.74%
	Myocardial Infarction/CAD	13	11.30%
	COPD	8	6.96%
	Dyslipidemia	7	6.09%
	Hypothyroidism	5	4.35%
	Atherosclerosis	4	3.48%
	Chronic Kidney Disease	3	2.61%
	Atrial fibrillation	1	0.87%

**Figure 1:**
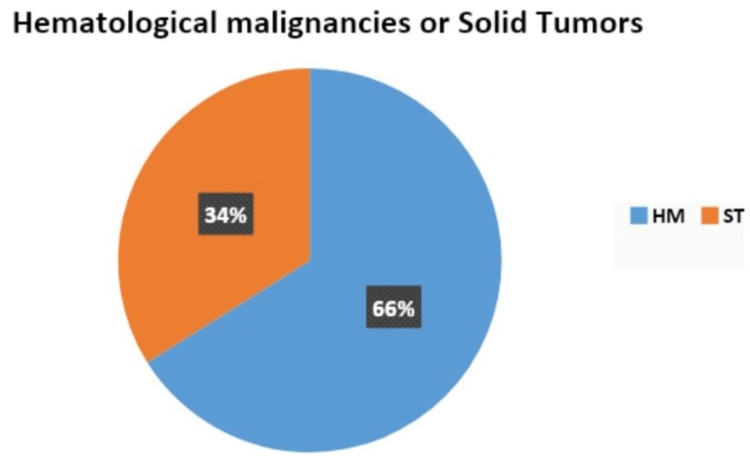
Distribution of hematological malignancies or solid tumors Sample size (n)= 115 HM: Hematological malignancies; ST: Solid tumors

There were more patients with hematological malignancies (66%) that presented with febrile neutropenia than solid tumor malignancies (34%), as evident from Figure 1. The distribution of the various malignancies is given in Tables 2, 3. The most common hematological malignancy was acute myeloid leukemia (48.68%), followed by non-Hodgkin lymphoma (30.26%). The most common solid tumor was osteosarcoma (17.95%), followed by carcinoma breast (12.82%) and carcinoma lung (12.82%).

**Table 2:** Distribution of type of cancer among hematological malignancies HM: Hematological malignancies; n (%): Number of cases (Percentage); Sample size (N): 76

Type of cancer	HM n(%) [N=76]
Acute Myeloid Leukemia	37 (48.68%)
Non-Hodgkin Lymphoma	23 (30.26%)
Hodgkin Lymphoma	6 (7.89%)
Acute Lymphoblastic Leukemia	4 (5.26%)
Myelodysplastic Syndrome	3 (3.95%)
Hairy Cell Leukemia	2 (2.63%)
Multiple Myeloma	1 (1.32%)

**Table 3:** Distribution of type of cancer among solid tumors ST: Solid tumors; n%= Number of cases (percentage); Sample size (N)= 39

Type of cancer	ST n(%) [N=39]
Osteosarcoma	7 (17.95%)
Breast cancer	5 (12.82%)
Carcinoma Lung	5 (12.82%)
Carcinoma prostate	3 (7.69%)
Colon cancer	2 (5.13%)
Germ cell tumor	2 (5.13%)
Medulloblastoma	2 (5.13%)
Pancreatic cancer	2 (5.13%)
Retroperitoneal Sarcoma	2 (5.13%)
Astrocytoma	1 (2.56%)
Carcinoma Rectum	1 (2.56%)
Carcinoma Endometrium	1 (2.56%)
Carcinoma Ovary	1 (2.56%)
Carcinoma tongue	1 (2.56%)
Cholangiocarcinoma	1 (2.56%)
Dysgerminoma Uterus	1 (2.56%)
Pleomorphic Sarcoma	1 (2.56%)
Rhabdomyosarcoma	1 (2.56%)

The distribution of the degree of neutropenia is shown in Table 4. Mild neutropenia (defined as an absolute neutrophil count (ANC) of < 1,500 cells/mm^3^ and <,1000 cells/mm^3^) was seen in five patients (4.3%), moderate neutropenia (defined as a count >1,000 cells/mm^3^ and >500 cells/mm^3^ ) was seen in 13 patients (11.3%), Severe neutropenia (defined as < 500 cells/mm^3^ and >100 cells/mm^3^ ) was seen in 42 patients (36.5%), and profound neutropenia (defined as <100 cells/mm^3^) was seen in 55 patients (47.8%). The range of neutropenia was from 0 to 1420, with a median of 318 cells/mm^3^. Overall pancytopenia was seen in 68 cases (59.13%)

**Table 4:** Distribution of severity of neutropenia among the study population Sample size (N)= 115

Severity of Neutropenia	Counts	Percentage
Mild (1001-1500 cells/mm^3^)	5	4.30%
Moderate (501-1000 cells/mm^3^)	13	11.30%
Severe (101-500 cells/mm^3^)	42	36.50%
Profound (<100 cells/mm^3^)	55	47.80%
Total	115	100%

Microbiological profile

A total of 115 cultures were analyzed. The distribution of the cultures taken has been shown in Table 5. Most of these cultures (93%) were blood cultures. Other sites of culture were pus (2.61%), urine (2.61%), sputum (0.87%), and broncho-alveolar lavage (0.87%). In the blood cultures, most cultures were drawn from a peripheral poke (87.85%). Other sites for drawing blood cultures were peripherally inserted central catheter (PICC) lines (7.47%), central lines (2.80%), and chemoport (1.87%). From all the cultures taken from the 115 cases (Figure 3), only 50 cultures (43%) showed microbial growth. As represented in Table 6, most of the cultures grew Gram-negative bacteria (88%), whereas only 8% grew Gram-positive bacteria, and 4% grew Candida, which showed no gram staining. A total of 12 cultures (24%) showed polymicrobial growth with more than one organism.

**Table 5:** Assessment of culture taken among the study population Sample size (n)= 115

Culture taken	Counts	Percentage
Blood	107	93.04%
Pus	3	2.61%
Urine	3	2.61%
Sputum	1	0.87%
Broncho-Alveolar Lavage	1	0.87%
Total	115	100%
Site of culture taken (If blood)		
Peripheral poke	94	87.85%
PICC line	8	7.475
Central line	3	2.80%
Chemoport	2	1.87%

**Table 6:** Gram staining of organisms isolated Sample size (n)= 50

Gram stain	Count	Percentage
Gram-positive	4	8%
Gram-negative	44	88%
Not applicable (fungi)	2	4%

**Figure 2:**
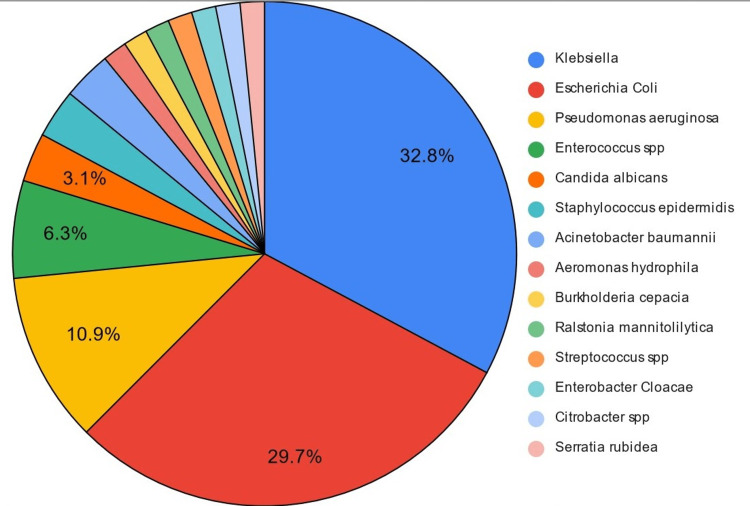
Microbiological profile Sample size (n)= 64

Across the 50 cultures that showed microbial growth, a total of 64 cases of microorganisms were isolated. From the cultures that showed growth (Table 7 and Figure 2), the most common organism isolated was *Escherichia coli*, which grew in 19 cultures (29.7%), followed by *Klebsiella pneumoniae*, which grew in 21 cultures (32.8%). *Pseudomonas aeruginosa* growth was seen in seven cultures (10.9%). *Enterococcus* spp was isolated in four cultures (6.25%). Other organisms that were also isolated were *Candida albicans* (3.13%), *Acinetobacter baumannii* (3.13%), *Staphylococcus epidermidis* (3.13%), *Streptococcus* spp (1.56%), *Enterobacter cloacae* (1.56%), *Burkholderia cepacia* (1.56%), *Aeromonas hydrophilia* (1.56%), *Serratia rubidea* (1.56%), *Citrobacter* spp (1.56%), and *Ralstonia mannitolytica* (1.56%).

**Table 7:** Distribution of specific growth observed among the cultures Sample size (n)= 64

Specification of growth	Frequency	Percentage
Klebsiella	21	32.81%
Escherichia Coli	19	29.69%
Pseudomonas aeruginosa	7	10.94%
*Enterococcus* spp	4	6.25%
Candida albicans	2	3.13%
Staphylococcus epidermidis	2	3.13%
Acinetobacter baumannii	2	3.13%
Aeromonas hydrophila	1	1.56%
*Burkholderia *cepacia	1	1.56%
Ralstonia mannitolilytica	1	1.56%
*Streptococcus *spp	1	1.56%
Enterobacter Cloacae	1	1.56%
*Citrobacter* spp	1	1.56%
Serratia rubidea	1	1.56%
Total	64	100%

The prevalence of multi-drug resistant organisms was found to be 32.8%, whereas the prevalence of extensively drug resistant organisms was found to be 18.75%. The prevalence of sensitive organisms was found to be 48.45%.

When observed based on microorganisms, in *Escherichia coli*, only 15.79% of cases were found to be sensitive while 68.42% of cases were found to be MDR with 15.79% cases of XDR organisms. *Klebsiella* showed a prevalence of 47.62% cases of sensitive cultures, while 14.28% of the cultures were MDR and 38.09% of the cases were XDR organisms. *Pseudomonas aeruginosa* showed 85.71% sensitive cultures and 14.28% XDR cultures. Both the candida cultures that grew were sensitive to all antifungals. 

*Aeromonas hydrophila*, *Burkholderia cepacian*, *Streptococcus* spp, *Acinetobacter baumannii*, *Citrobacter *spp, and *Serratia rubidea* cultures were all sensitive to antimicrobials. Interestingly, for the two cultures of *Staphylococcus* identified, one was found to be methicillin-resistant whereas the other was found to be methicillin sensitive.

Looking at each antibiotic specifically (Table 8), it is seen that most organisms show resistance to amoxicillin-clavulanic acid, ceftriaxone, levofloxacin, and ciprofloxacin. In *E. coli*, most isolates showed resistance with amoxicillin-clavulanic acid (11.11% susceptible) followed by ciprofloxacin (16.67% susceptible), levofloxacin (16.67% susceptible), and ceftriaxone (16.67% susceptible), whereas sensitivity was still maintained for amikacin, meropenem and piperacillin-tazobactam. In *Klebsiella pneumoniae*, most isolates showed resistance to amoxicillin-clavulanic acid (23.52% susceptible), levofloxacin (23.53% susceptible), and ciprofloxacin 23.53% susceptible). *Klebsiella* showed susceptibility rates of less than 55% for all antibiotics tested in the sensitivity panel.

**Table 8:** Specific antibiotic susceptibility distribution

Organism	Piptaz (%)	Ciprofloxacin (%)	Amikacin (%)	Gentamicin (%)	Meropenem (%)	Cefepime (%)	Ceftriaxone (%)	Amoxiclav (%)	Levofloxacin (%)	Aztreonam (%)
E. Coli	77.78	16.67	88.89	77.78	83.33	22.22	16.67	11.11	16.67	-
Klebsiella	52.95	23.53	52.95	47.05	52.95	35.29	35.29	23.53	23.53	53
Pseudomonas	60	80	80	80	80	60	80	-	80	-

Outcomes

Of the 115 cases admitted for febrile neutropenia, 33.92% (39) of the cases required ICU stay, which consisted of 27 cases that were hematological malignancies and 12 cases that were solid tumors. When compared to the number of patients admitted for febrile neutropenia, the ICU admission rate according to the type of malignancy was 35.52% for hematological malignancies and 30.76% for solid tumors. The median duration of ICU stay was three days, ranging from 1 day to 30 days. Intubation had to be done in 14 cases (12.17%), out of which nine cases were in hematological malignancies while five were solid tumors. Of all cases of febrile neutropenia seen in the hospital, 19 cases (16.52%) ended in death with 13 cases of hematological malignancies and six cases of solid tumors. Thus mortality rate was 17.11% in hematological malignancies and 15.38% in solid tumors.

**Table 9:** Outcomes of admission Sample size (n)= 115

Outcomes	Yes	No
Mortality	19 (16.52%)	96 (83.47%)
ICU stay	39 (33.92%)	76 (66.08%)
ICU stay duration		
Range	1 to 30 days	
Median	3 days	

## Discussion

Febrile neutropenia continues to be a major cause of morbidity, mortality, increasing healthcare costs, and decreased efficacy of treatment resulting from delays and decreased doses [12]. Comparisons can be made with similar studies conducted in other centers [7,9,19-23]. Most studies on febrile neutropenia were done only on patients with hematological malignancies [23,24] with only a few studies being conducted on a population consisting of both hematological and solid tumors [20]. Culture positivity rates in our institution seemed to be slightly higher than what we found in other institutes during our literature review. Most studies showed a culture positivity rate in the range of 17-34% [6,7,19,20]. However, Swati et al. reported a positivity rate of 41.2% [23] which was comparable to what we found in our study.

Febrile neutropenia can be caused by any microorganism, from bacteria to fungi to viruses [7]. While Gram-negative bacteria were more frequently isolated from cultures, it was unusual how few of the cultures grown had isolated Gram-positive organisms. An overwhelming majority (88%) of cultures isolated Gram-negative organisms, while only a few (4%) cultures isolated Gram-positive organisms. *Klebsiella pneumoniae* was the most common (32.81%) organism isolated followed by *Escherichia coli* (29.69%), as has been seen in similar studies [19-24]. However surprisingly, *Staphylococcus aureus*, *Staphylococcus epidermidis, *and *Streptococcus* were isolated in very few cultures (0%, 3.13%, and 1.56% respectively). While there had been a trend of cultures isolating Gram-positive bacteria in the past during the 90s and 00s [22] as described by Wisplinghoff et al. [5] and Viscoli and Castagnola [4], newer studies have been showing a growing trend of the increasing prevalence of Gram-negative bacteria being isolated, as was noted by Jacob et al. [20] and Hakim et al. [7] among others [3,23,25].

Swati et al. and Jacob et al. reported a mortality rate of 20.3% and 13.3% respectively in febrile neutropenia patients from India [20,23]. Mortality rates from febrile neutropenia in our hospital were found to be 16.52%, which is consistent with these numbers. We also found that mortality rates were similar in hematological malignancies and solid tumors.

Another disturbing trend was the increased instances of MDR and XDR organisms. While the majority of organisms remained sensitive to antibiotics, a large number of organisms had begun developing multi-drug resistance and extensive-drug resistance. In *E. coli* isolates, more than 80% of the isolates were resistant to at least two classes of antibiotics with 68% of the isolates showing multi-drug resistance and 15% being extensively drug resistant. In *Klebsiella*, more than half the isolates showed resistance to at least two classes of antibiotics which was concerning as this leads to decreased antibiotic options. The data showed an alarmingly high resistance rate in *Klebsiella* isolates to all antibiotic options available. *Pseudomonas* was thankfully still predominantly sensitive to antibiotics although this trend could change in the future. This can be attributed to the rampant and improper use of antibiotics throughout the healthcare industry leading to the rise of antibiotic-resistant organisms. If left unchecked, this could decrease the options available to treat even common organisms. The emergence of resistance to multiple antimicrobial agents in pathogenic bacteria has become a significant public health threat as there were fewer, or even sometimes no, effective antimicrobial agents available for infections caused by these bacteria. Gram-positive and Gram-negative bacteria are both affected by the emergence and rise of antimicrobial resistance [16].

Studies show worrying trends of increased carbapenem resistance and cephalosporin resistance in Gram-negative bacteria. Colistin has been reintroduced to try and combat antimicrobial resistance, but colistin resistance has also begun slowly appearing. Aminoglycosides are an antibiotic class used for both Gram-positive and Gram-negative organisms and this class of antibiotics is usually used as second-or third-line agents in the treatment of infectious diseases including methicillin-resistant *Staphylococcus aureus* (MRSA) and multidrug-resistant tuberculosis (MDR-TB) [26]. Hence, the development of resistance towards aminoglycoside is considered to be very alarming. Research has been directed towards producing newer aminoglycoside and as an example, plazomicin was developed with improved activity against the resistant strains of MDR Gram-negative bacteria and MRSA [26,27].

One major drawback of our study was the small number of reported cases. A solution to this would be conducting multi-hospital observational studies to obtain higher numbers. Another drawback was that only the first culture that showed growth was considered for recording the microbial infection in patients with multiple cultures taken. Subsequent cultures were included only if they showed the growth of a new organism. This could lead to a possibly altered view of the disease profile in patients who may have had re-infections with the same organism or co-infections.

## Conclusions

With the changing flora and susceptibility pattern to antibiotics, new guidelines to determine empirical therapy for infections in neutropenic patients must be identified. Our study can be used along with similar studies done in other institutes to develop a whole picture of the epidemiology of febrile neutropenia in the country. It can also be used to tailor antibiotics administered in the hospital by identifying current trends of microbiological antibiotic sensitivity to help decrease mortality and morbidity from febrile neutropenia.
